# FAK activation is required for IGF1R-mediated regulation of EMT, migration, and invasion in mesenchymal triple negative breast cancer cells

**DOI:** 10.18632/oncotarget.3023

**Published:** 2015-02-28

**Authors:** LaTonia Taliaferro-Smith, Elaine Oberlick, Tongrui Liu, Tanisha McGlothen, Tiffanie Alcaide, Rachel Tobin, Siobhan Donnelly, Rachel Commander, Erik Kline, Ganji Purnachandra Nagaraju, Lauren Havel, Adam Marcus, Rita Nahta, Ruth O'Regan

**Affiliations:** ^1^ Department of Hematology and Medical Oncology, Winship Cancer Institute, Emory University School of Medicine, Atlanta GA 30322 USA; ^2^ Graduate Program in Biological and Biomedical Sciences, Harvard Medical School, Boston, MA, 02115 USA; ^3^ Department of Pharmacology, Emory University School of Medicine, Atlanta, GA 30322 USA; ^4^ Georgia Cancer Center for Excellence at Grady Memorial Hospital, Atlanta, GA, 30303 USA

**Keywords:** Triple-negative breast cancers (TNBC), insulin-like growth factor 1 receptor (IGF1R), focal adhesion kinase (FAK), epithelial-mesenchymal transition (EMT), invasion

## Abstract

Triple negative breast cancer (TNBC) is a highly metastatic disease that currently lacks effective prevention and treatment strategies. The insulin-like growth factor 1 receptor (IGF1R) and focal adhesion kinase (FAK) signaling pathways function in numerous developmental processes, and alterations in both are linked with a number of common pathological diseases. Overexpression of IGF1R and FAK are closely associated with metastatic breast tumors. The present study investigated the interrelationship between IGF1R and FAK signaling in regulating the malignant properties of TNBC cells. Using small hairpin RNA (shRNA)-mediated IGF1R silencing methods, we showed that IGF1R is essential for sustaining mesenchymal morphologies of TNBC cells and modulates the expression of EMT-related markers. We further showed that IGF1R overexpression promotes migratory and invasive behaviors of TNBC cell lines. Most importantly, IGF1R-driven migration and invasion is predominantly mediated by FAK activation and can be suppressed using pharmacological inhibitors of FAK. Our findings in TNBC cells demonstrate a novel role of the IGF1R/FAK signaling pathway in regulating critical processes involved in the metastatic cascade. These results may improve the current understanding of the basic molecular mechanisms of TNBC metastasis and provide a strong rationale for co-targeting of IGF1R and FAK as therapy for mesenchymal TNBCs.

## INTRODUCTION

Triple-negative breast cancers (TNBCs) are distinguished by the absence of estrogen receptor (ER), progesterone receptor (PR), and human epidermal growth factor receptor 2 (HER2) expression and constitutes up to 20% of all breast cancers cases. TNBCs account for a disproportionate number of deaths from breast cancer, especially among premenopausal African-American and Hispanic women. High mortality rates among TNBC patients can partly be attributed to a propensity to develop distant metastases within 5-years of diagnosis, despite the use of systemic chemotherapy. The mechanisms underlying this propensity for metastases noted with TNBC remain poorly understood. Unlike other subtypes of breast cancer, there are currently no targeted agents approved to treat TNBCs, and the only option for patients is systemic chemotherapy with its inherent toxicities. Understanding mechanisms underlying the metastatic potential of TNBC can aid in the development of novel therapies that reduce the number of deaths linked to this breast cancer subtype.

The IGF1R signaling cascade has received increased attention as a potential therapeutic target for breast cancers. Overexpression of IGF1R is common in breast carcinomas, and xenograft studies demonstrate that IGF1R up-regulation induces mammary tumor growth and metastases [1]. Furthermore, using genetic ablation of IGF1R expression in a basal-like breast cancer xenograft model, it has been shown that IGF1R is involved in breast tumorigenesis [2]. Elevated IGF1R levels appear to enhance cell survival and metastasis following chemotherapy, potentially leading to decreased survival for breast cancer patients [3]. Furthermore, phosphorylated IGF1R is detected in nearly 42% of TNBCs and is associated with poor survival [4]. There is some evidence showing differential effects of cytoplasmic IGF1R expression on disease-free survival (DFS) and breast cancer specific survival (BCSS) in ER-positive versus ER-negative breast cancers [5]. Elevated levels of IGF1R in ER-positive breast cancers correlated strongly with DFS and BCSS, whereas expression of IGF1R was associated with shorter DFS in TNBCs [5].

Several studies have provided new insights into the biological effects of insulin-like growth factor 1 (IGF-1) ligand in epithelial to mesenchymal transition (EMT) in breast cancers [6–10]. For example, IGF-I induces the migration of breast cancer cells and increases the expression of genes involved in EMT [8, 9]. Taken together, these data suggest a key role for the IGF1R signaling axis in the metastatic nature of TNBCs and warrant further investigation into the molecular mechanisms regulated by IGF1R.

Given the importance of IGF1R signaling in carcinogenesis, a number of pharmacological antagonists have been developed. Several IGF1R/IR-specific small molecule inhibitors and human monoclonal antibodies are currently at various stages of preclinical and clinical investigation (reviewed in [11]). However, the majority of clinical trials have not produced encouraging results to date. This may be because available agents do not effectively inhibit IGF1R signaling or because of compensatory mechanisms that negate the effects of single therapies.

Focal adhesion kinase (FAK) is a cytoplasmic non-receptor tyrosine kinase whose activation by integrins leads to activation of signaling mechanisms that promote tumorigenesis. Increased FAK activity has been detected in several invasive and metastatic cancer tissues, including breast, thyroid, prostate, and head and neck tumors, but is undetectable in normal and benign tumor tissues [12–16]. FAK gene amplification and overexpression has been demonstrated in primary and invasive metastatic breast cancers and may be a biomarker for invasive potential of breast tumors [17]. Several downstream signaling cascades have been shown to mediate FAK-induced cell migration, including Src and PI3K [18]. Cytoskeletal reorganization, cell motility, and local invasion of the host tissue are important factors in cancer metastasis. FAK plays a central role in regulating cell adhesion, polarity and motility by coordinating the spatiotemporal changes in the actin cytoskeleton and by activating Rac, Rho, and cdc42 GTPases [19–22], a family of proteins which are important regulators of cell polarity events like lamellipodia formation and Golgi reorientation.

Co-immunoprecipitation and degradation studies demonstrated that FAK directly binds to, stabilizes, and activates IGF1R in human cancer cells [23]. Zheng et al. demonstrated that the FERM domain of FAK directly binds to the β-subunit of IGF1R, which contains the tyrosine kinase domain [24]. Down-regulation of FAK results in degradation of IGF1R, and dual inhibition of FAK and IGF1R produces synergistic anti-tumor effects [25, 26]. Furthermore, co-inhibition of FAK and IGF1R decreased human glioma cell survival, increased cell detachment, and induced apoptosis via increased caspase-3 and PARP cleavage [26]. Hence, we hypothesized that crosstalk between the IGF1R and FAK pathways may be a mechanism by which IGF1R affects EMT, invasion, and metastatic processes in TNBCs. Therefore, we sought to determine the precise roles of these molecules in TNBC migration and invasion of metastatic TNBC cell lines.

In this study, we specifically investigated the functions of IGF1R and FAK signaling and their involvement in the metastatic potential of human TNBCs. We show that IGF1R is a key regulator of EMT in human TNBC cells, and most interestingly, inhibition of IGF1R results in MET in TNBC cells of mesenchymal-like origin. Ablation of IGF1R results in decreased colony formation, migration, and invasion of TNBC cells. We further demonstrate that IGF1R mediates these processes in TNBC cells via the IGF1R/FAK signaling pathway. These observations demonstrate that IGF1R may promote TNBC metastasis in a FAK-dependent manner and provide a solid basis for exploring co-targeting IGF1R and FAK as a potential therapeutic approach in a subset of mesenchymal-like TNBCs.

## RESULTS

### IGF1R and FAK expression levels and validation of stable IGF1R knockdown in TNBC cells

**Figure 1:**
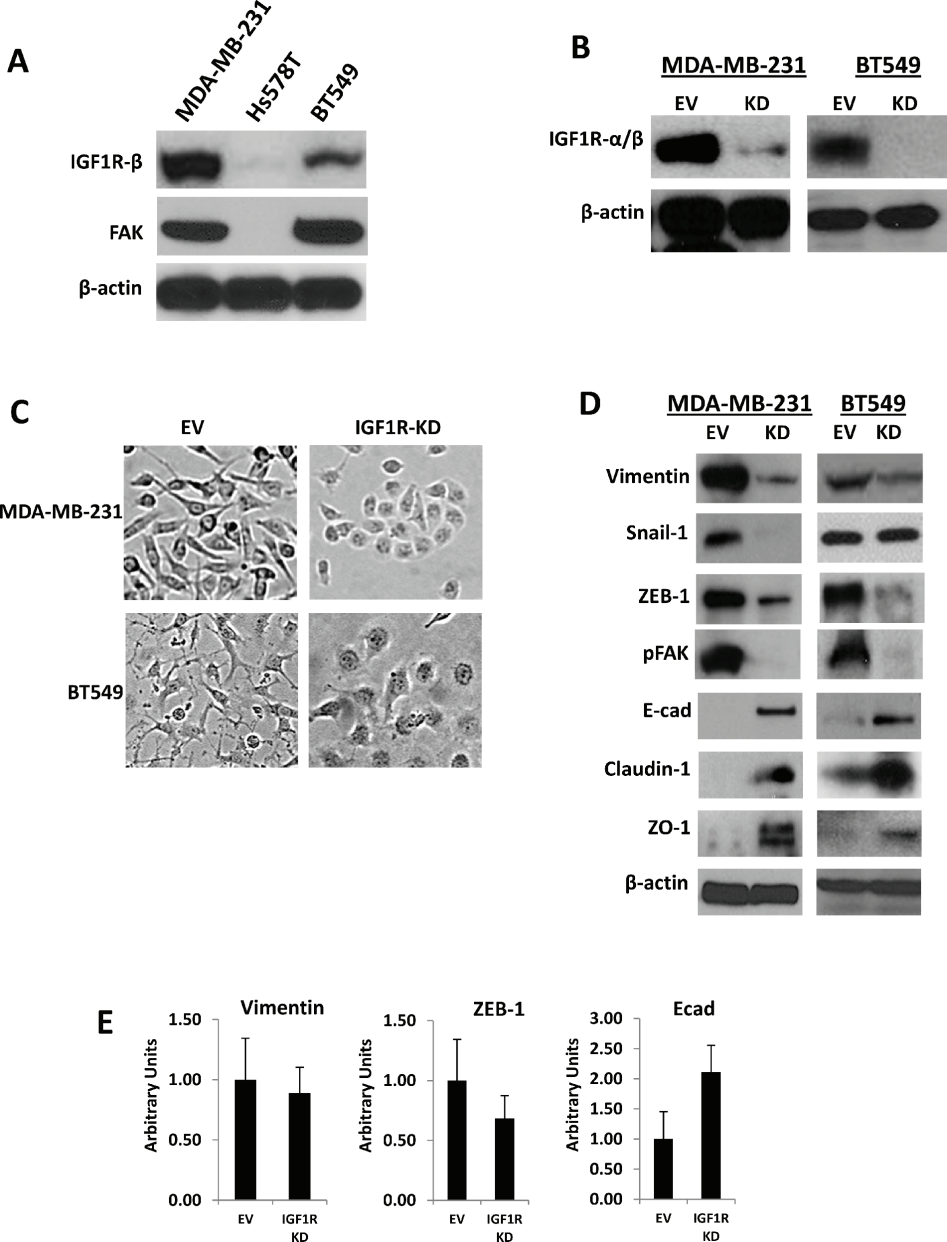
Stable silencing of IGF1R confers epithelial-like phenotypes in mesenchymal TNBC cells **(A)** Endogenous expression of IGF1R-β and total FAK analyzed via Western blot analysis in a panel of mesenchymal human TNBC cells. **(B)** Western blot confirmation of stable lentiviral knockdown of IGF1R-α/β (IGF1R-KD) in MDA-MB-231 and BT549 TNBC cells. β-actin was used as a loading control. **(C)** Morphological changes in MDA-MB-231 and BT549 IGF1R-KD cells compared to EV control cells four to six passages post-lentiviral infections; brightfield magnification x20. **(D)** Western blot analyses of mesenchymal markers (vimentin, Snail-1, ZEB-1), motility marker pFAK, and epithelial markers (E-cadherin, claudin-1, and ZO-1) in cell lines stably expressing EV control plasmid or IGF1R-KD lentiviral plasmids using specific antibodies. β-actin was used as a loading control. **(E)** Relative mRNA expression levels of Vimentin, ZEB-1, and E-cadherin in MDA-MB-231 EV and IGF1R-KD cell lines was detected by TaqMan quantitative RT-PCR and normalized to RPLPO. The relative amounts of transcript were described using the 2–ΔΔCt method. Data are displayed in means ± standard deviation of at least three independent experiments of each group.

Elevated expression of IGF1R was previously reported in tumor samples of patients with ER-negative breast cancers (5). To obtain evidence of expression in cultured TNBC cells, IGF1R and FAK protein levels were determined by Western blotting in a panel of mesenchymal human TNBC cell lines (MDA-MB-231, Hs578T, and BT-549) (Figure 1A). BT-549, MDA-MB-231, and Hs578T cells have been classified as MSL/M subtypes with mesenchymal characteristics [27]. Both MDA-MB-231 and BT549 TNBC cells expressed IGF1R and FAK protein, while Hs578T cells expressed undetectable levels of both IGF1R and FAK protein (Figure 1A). To determine the role of IGF1R in TNBC cells, we established lentivirus-mediated stable TNBC anti-IGF1R-α/β (IGF1R-KD) and empty vector (EV) control cell lines. Immunoblotting analysis showed that the expression level of IGF1R in MDA-MB-231/IGF1R-KD and BT549/IGF1R-KD cells was reduced compared to EV control cells (Figure 1B), suggesting that lentivirus infection was highly efficient at inhibiting IGF1R expression.

### Loss of IGF1R in mesenchymal TNBC cells promote epithelial-like phenotypes

Interestingly, we noted that knockdown of IGF1R induced mesenchymal to epithelial transition (MET) in TNBC cells displaying mesenchymal characteristics. Morphologically, MDA-MB-231 and BT549 EV control cells were spindle-shaped mesenchymal cells, whereas MDA-MB-231 and BT549 IGF1R-KD cells were tightly bound, rounded cells with epithelial phenotypes (Figure 1C). Moreover, these observations were confirmed by Western blotting using antibodies specific for EMT-related markers, including mesenchymal (vimentin, Snail-1, and ZEB-1) and epithelial (E-cadherin, claudin-1, and ZO-1) markers. As shown in Figure 1D, consistent with their cobblestone-like, epithelial morphologies, vimentin, Snail-1, and ZEB-1 protein levels were down-regulated and E-cadherin, claudin-1, and ZO-1 protein expressions were up-regulated in IGF1R-KD cells compared to controls. Quantitative real-time PCR also revealed decreased mRNA expression of vimentin and ZEB-1 and increased expression of E-cadherin mRNA levels in stable MDA-MB-231 IGF1R-KD cells relative to EV controls (Figure 1E). Based on these morphologic changes shown in IGF1R-null cells, we concluded that IGF1R plays an essential role in inducing EMT-like phenotypes in TNBC cells, which renders them more motile and invasive.

### Stable IGF1R inhibition suppresses TNBC cell colony formation, migration, and invasion

**Figure 2:**
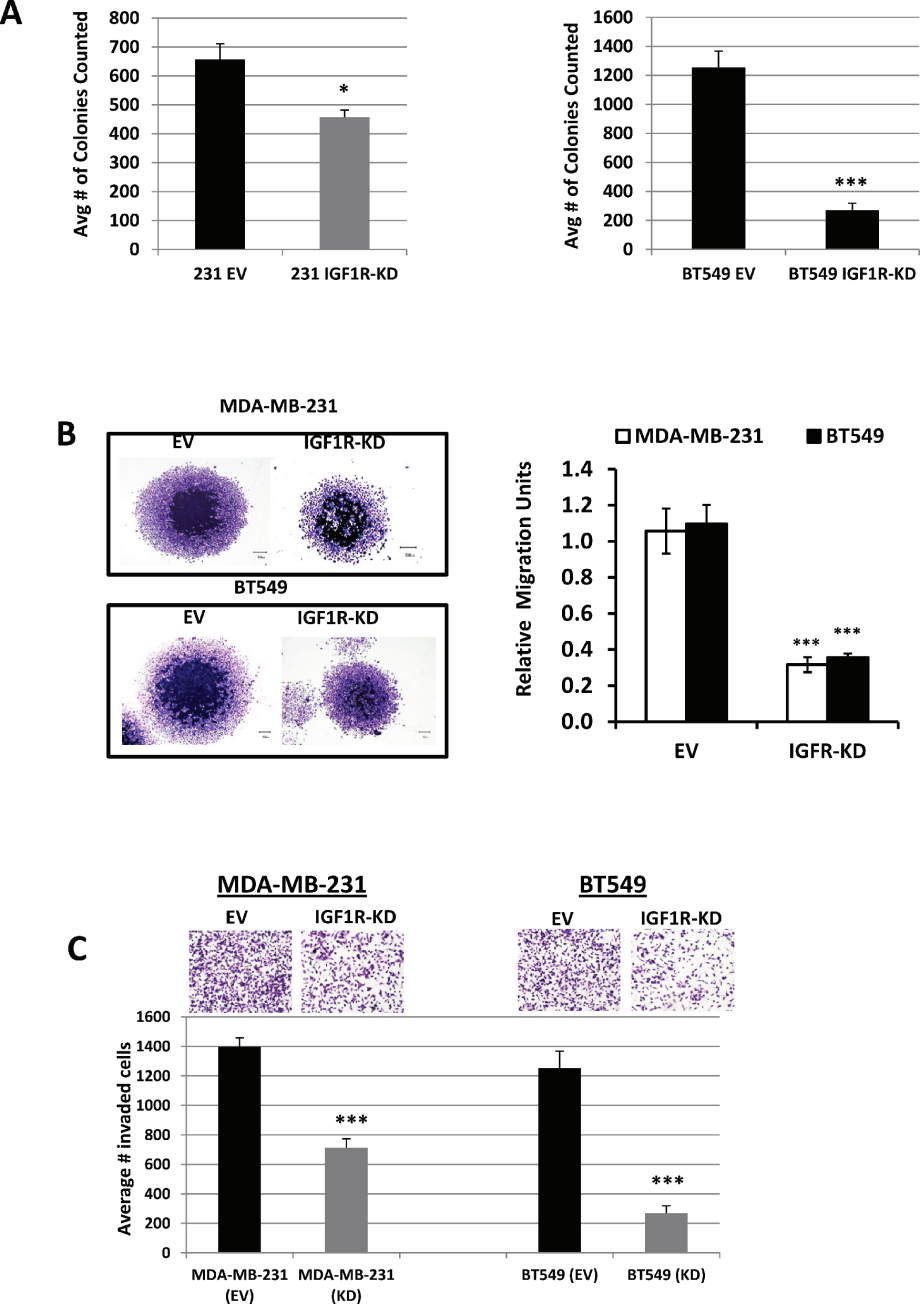
Inhibition of IGF1R suppresses TNBC cell colony formation, migration, and invasion **(A)** Colony formation assays using MDA-MB-231 and BT549 EV-control and IGF1R-KD cells; colonies counted contained at least > 50 cells/colony. Data are representative of the average of at least three independent experiments performed in triplicate. *p = 0.042 and ***p < 0.001 compared to EV control cells. **(B)** Evaluation of *in vitro* cell migration potentials of MDA-MB-231 and BT549 EV-control and IGF1R-KD cells by spheroid migration assay. Representative images (left, magnification x20) and the mean relative migration (±S.D.) in five different spheroids (right) are shown. ***p < 0.001 compared to EV control cells. **(C)** Representative images of cell invasion assays of MDA-MB-231 and BT549 EV control and IFG1R-KD cells plated in the upper chambers of Transwell units coated with Matrigel. Fetal bovine serum and fibronectin was used as chemo-attractants in the lower chambers. The results are expressed as the average number of invaded cells per field of view (means ± S.D.; *n* = 6). ***p < 0.001 compared to EV control cells.

Cell migration and invasion are important hallmarks of malignant cells that contribute to metastatic phenotypes. In the present study, we assessed the effects of IGF1R inhibition on TNBC clonogenecity, cell migration, and invasion using colony formation, spheroid migration and Matrigel invasion assays, respectively. As expected, knockdown of IGF1R resulted in a significant decrease in colony growth of MDA-MB-231 (1.4-fold change; *p* = 0.042) and BT549 (4.4-fold change; *p* < 0.001) cells compared with EV control cells (Figure 2A). Because tumor spheroids mimic tumor migratory characteristics, we formed MDA-MB-231 and BT549 IGF1R-KD spheroids and compared these results to the EV control groups. Our results show a significantly higher radial migration patterns in EV controls as compared to IGF1R-KD cell lines (*p* < 0.001) (Figure 2B). These results clearly demonstrate the involvement of IGF1R in the migratory capabilities of TNBC cells. We next performed Matrigel invasion assays to examine the effects of IGF1R down-regulation on the invasive potential of TNBC cells. As evident from Figure 2C, IGF1R inhibition significantly decreased invasion of both MDA-MB-231 and BT549 IGF1R-KD cells compared to EV control cells (*p* < 0.001). Collectively, these results show that IGF1R inhibition effectively inhibits colony formation, migration, and invasion of mesenchymal TNBC cells.

### siRNA-mediated FAK down-regulation inhibits IGF1R expression and invasive potentials of TNBC cells

**Figure 3:**
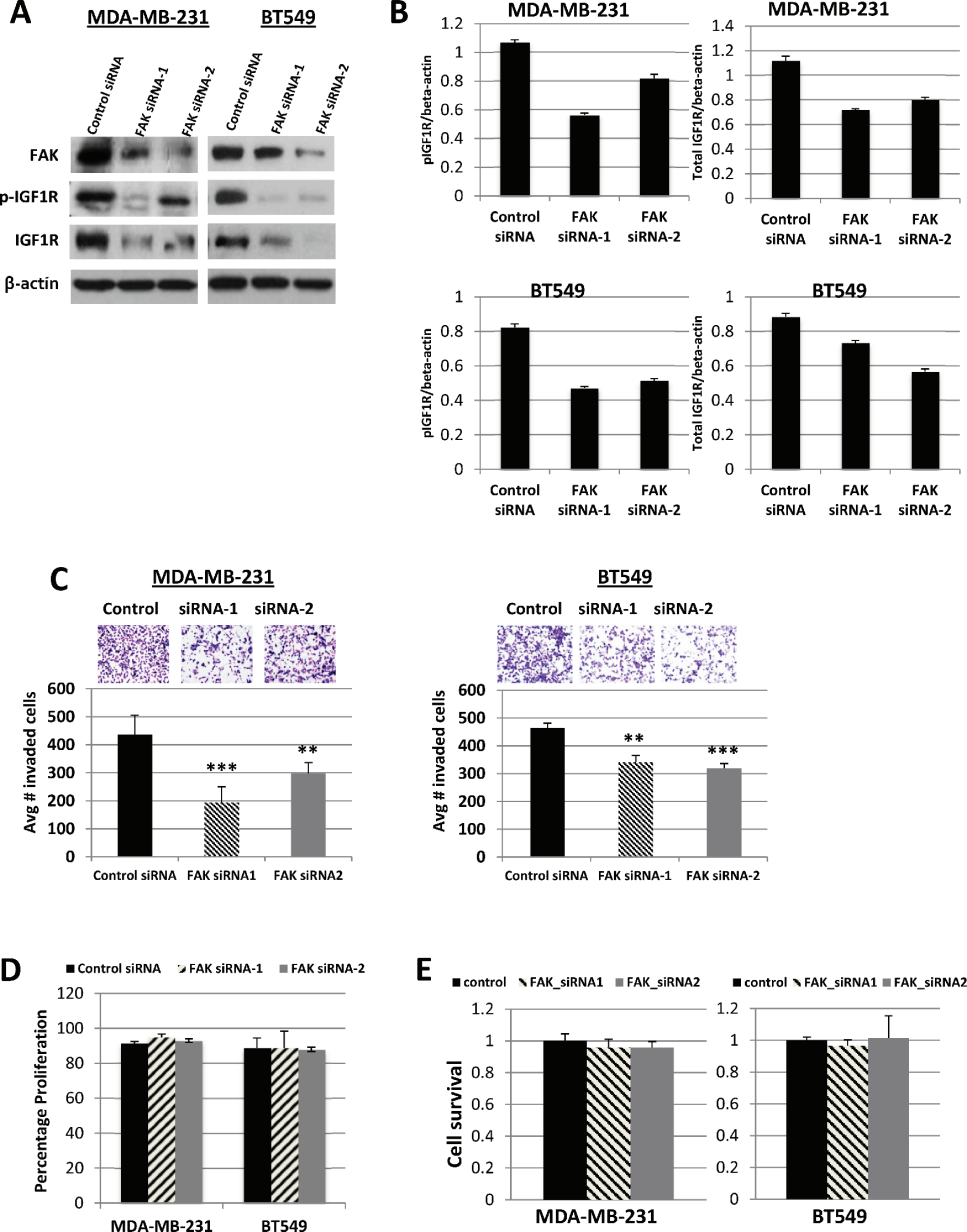
Effects of FAK siRNA silencing on IGF1R expression, and cell invasion, proliferation, and survival **(A)** Western blot analysis of FAK, pIGF1R, and total IGF1R protein levels in MDA-MB-231 and BT549 cells transiently transfected for 48 h with 50 nM of control siRNA, FAK siRNA-1, or FAK siRNA-2. β-actin was used as a loading control. The protein levels were confirmed in three independent experiments. **(B)** Quantified data of pIGF1R and total IGF1R protein levels, normalized to β-actin. Means and SDs of three separate experiments are shown. **(C)** Representative images of Transwell cell invasion assays of MDA-MB-231 and BT549 cells transiently transfected with 50 nM of control siRNA, FAK siRNA-1, or FAK siRNA-2. Pictures were taken at 20x magnification. The histograms show the average number of invasive cells (error bars represent S.D. of three independent experiments, each performed in replicates of five. **p < 0.01 and ***p < 0.001 compared to control siRNA transfected cells. **(D)** MDA-MB-231 and BT549 cells were transiently transfected with 50 nM of control siRNA, FAK siRNA-1, or FAK siRNA-2 as described above. After 48 h, cells were counted by trypan blue exclusion; data represented as a percentage of the control siRNA groups. The results represent the average of triplicated treatment groups performed at least three times with reproducible results. **(E)** Cell lines were transfected as described above for 48 h and cell survival was measured by SRB assays. The data represent mean growth inhibition compared to control siRNA treated cells for three independent experiments for each cell line.

Previous studies have shown that FAK regulates IGF1R stability and auto-phosphorylation in several human cancer cells [23, 28]. Based on our observation that phosphorylated FAK levels were decreased in response to IGF1R silencing (Figure 1D), we sought to determine if FAK also regulated IGF1R activity in TNBC cell lines. We found that in both MDA-MB-231 and BT549 cells, siRNA-mediated FAK silencing resulted in decreased FAK expression and down-regulation of active and total IGF1R (Figures 3A and 3B). Further, we examined the effect of FAK silencing on *in vitro* cell invasion. Using Matrigel invasion assays, we found that MDA-MB-231 and BT549 cells with transient FAK knockdown exhibited a significant reduction in invasion (*p* < 0.001) as compared with cells treated with control siRNA (Figure 3C). We further demonstrated that these observed effects on invasion were not the result of differences in proliferative potential (Figure 3D) or influences on cell survival (Figure 3E).

### Effects of FAK-specific pharmacological inhibitors on expression of EMT markers, migration, and invasion in TNBC cells

**Figure 4:**
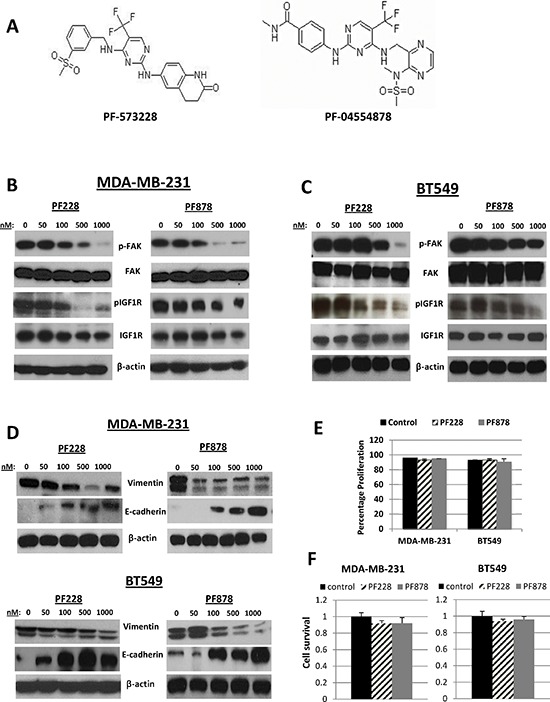
Effects of FAK-specific inhibitors on IGF1R activity, invasion, and EMT-related protein expression in TNBC cells **(A)** Chemical structures of two FAK tyrosine kinase inhibitors, PF-573228 and PF-04554878. The expression of pFAK, FAK, pIGF1R, and IGF1R were assessed via immunoblotting analysis in **(B)** MDA-MB-231 and **(C)** BT549 TNBC cells following treatments with indicated doses of PF228 and PF878 for 24 h. **(D)** Vimentin and E-cadherin expressions examined via Western blotting following treatments as indicated above. β-actin served as a loading control. **(E)** MDA-MB-231 and BT549 cells were treated with 0.5 μM PF228 or 0.5 μM PF878 for 24 h. Cells were counted by trypan blue exclusion; data represented as a percentage of the vehicle treated (DMSO) control groups. The results represent the average of triplicated treatment groups performed at least three times with reproducible results. **(F)** Cell lines were treated with 0.5 μM PF228 or 0.5 μM PF878 for 24 h and the effects on cell survival was measured by SRB assays. The data represent mean growth inhibition compared to vehicle treated (DMSO) control cells for three independent experiments for each cell line.

Next, we tested the phosphorylation status of FAK and IGF1R in TNBC cells after treatments for 24 h with increasing concentrations with FAK-specific inhibitors, PF228 and PF878 (also known as VS-6063 and defactinib) (Figure 4A). In MDA-MB-231 and BT549 cells, both inhibitors led to dose-dependent dephosphorylation of FAK on residue Y397, as well as IGF1R dephosphorylation at Y1135/Y1136 (Figures 4B and 4C), with the more pronounced decrease being observed following treatment with 0.5–1.0 μM inhibitor for 24 h. Interestingly, we noted that both PF228 and PF878 caused a decrease in vimentin and an increase in E-cadherin protein expression in a concentration-dependent manner (Figure 4D) with no apparent effects on cell proliferation (Figure 4E) or cell survival under the same treatment conditions (Figure 4F). These data demonstrate a reciprocal regulation of IGF1R by FAK and further confirm our findings that the IGF1R/FAK signaling cascade is involved in TNBC cell EMT.

**Figure 5:**
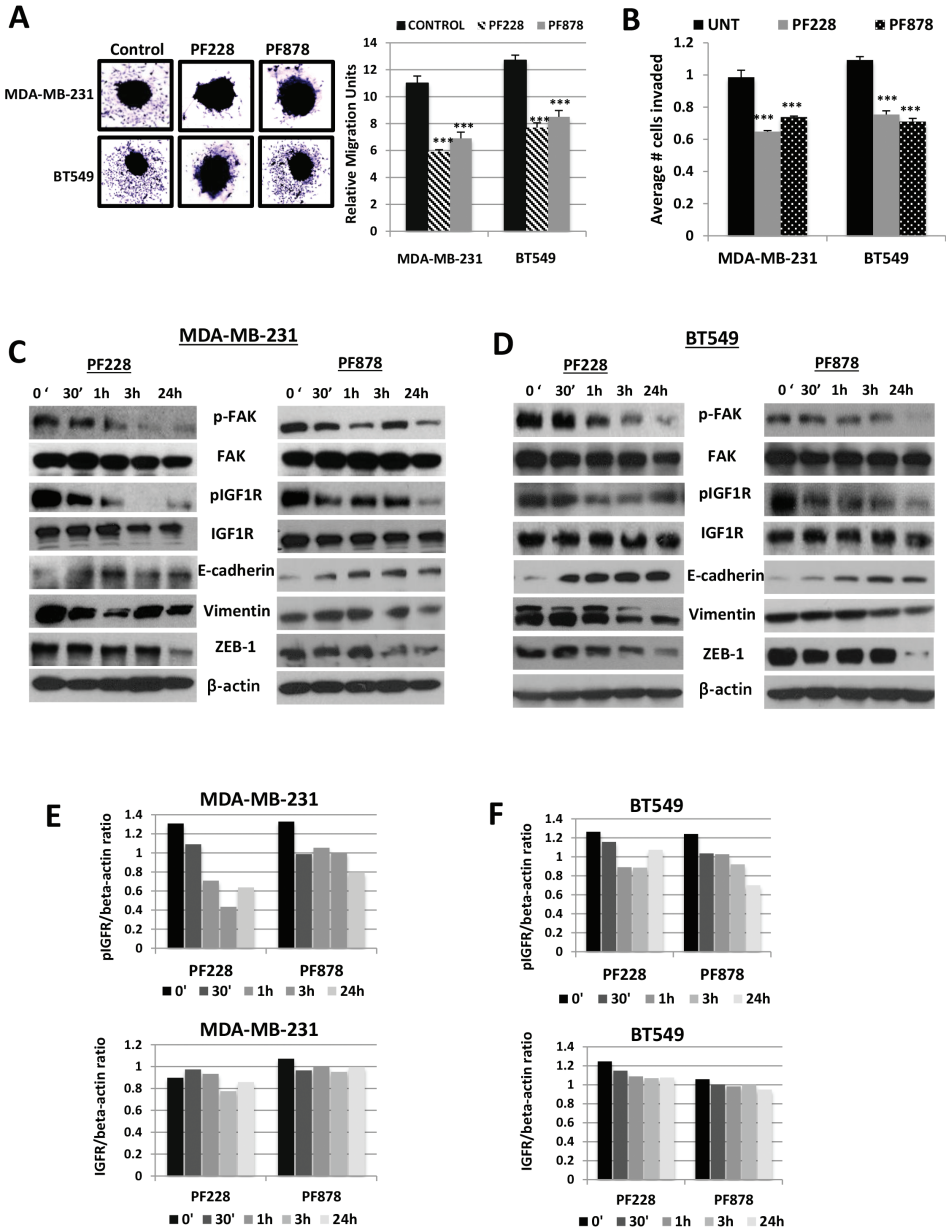
FAK-specific inhibitors suppress spheroid migration, decrease IGF1R phosphorylation, and alter EMT-marker expression levels in TNBC cells **(A)** Spheroid cell migration assays were performed to analyze the migration potential of MDA-MB-231 and BT549 cells treated for 24 h with vehicle control (DMSO), 0.5 μM PF228 or 0.5 μM PF878. Representative images (left, magnification x10) and the mean relative migration (±S.D.) of five different spheroids (right) are shown. ***p < 0.001 compared to untreated control cells. **(B)** Transwell cell invasion assays of MDA-MB-231 and BT549 cells treated with vehicle control (DMSO), 0.5 μM PF228, or 0.5 μM PF878 for 24 h. The histograms show the average number of invasive cells (error bars represent S.D. of three independent experiments, each performed in replicates of five). ***p < 0.001 compared to control cells. Total protein lysates from **(C)** MDA-MB-231 and **(D)** BT549 cells treated with vehicle control (DMSO), 0.5 μM PF228 or 0.5 μM PF878 for various time points (0, 30′, 1 h, 3 h, and 24 h) were analyzed via Western blotting for expression of pFAK, FAK, pIGF1R, IGF1R, E-cadherin, vimentin, and ZEB-1. β-actin served as a loading control. Quantified data of pIGF1R and total IGF1R levels, normalized to β-actin for **(E)** MDA-MB-231 and **(F)** BT549 cells. Means and SDs of three separate experiments are shown.

We performed spheroid migration assays to study whether FAK-specific tyrosine kinase inhibitors can influence migration of TNBC cells. MDA-MB-231 and BT549 spheroids grown in 96-well plates coated with 1% agarose were treated with vehicle control, 0.5 μM PF228, or 0.5 μM PF878. Twenty-four hours after treatments, the migrating capacity of control cells from spheroids was significantly higher than that of inhibitor-treated cells (*p* < 0.001) (Figure 5A). Furthermore, both FAK inhibitors significantly reduced the abilities of MDA-MB-231 and BT549 cell to invade across Matrigel-coated Boyden chambers (*p* < 0.001) (Figure 5B). Figure 5 demonstrates that phosphorylation of FAK at Y397 is decreased in a time-dependent manner in both cell lines, with a more pronounced decrease being observed following treatment with 0.5 μM PF228 or PF878 for 24 h. We also examined phosphorylated IGF1R levels in each cell line (Figures 5C–5F). Similar to decreased FAK activity, active IGF1R levels reduced in a time-dependent manner in both cell lines. Both total FAK and IGF1R protein expression levels remained unchanged. Most notably, vimentin protein levels were also reduced in a time-dependent (Figures 5C and 5D) manner following pharmacologic inhibition. E-cadherin protein levels were increased while its transcriptional repressor, ZEB-1 was inversely decreased in inhibitor-treated cells compared to untreated control cells (Figures 5C and 5D). These results further indicate that a major effect of targeting FAK signaling in TNBC cells is the suppression of IGF1R activity, EMT, migration, and invasion.

### FAK activation plays an essential role in IGF1R-induced migration and invasion of TNBC cells

**Figure 6:**
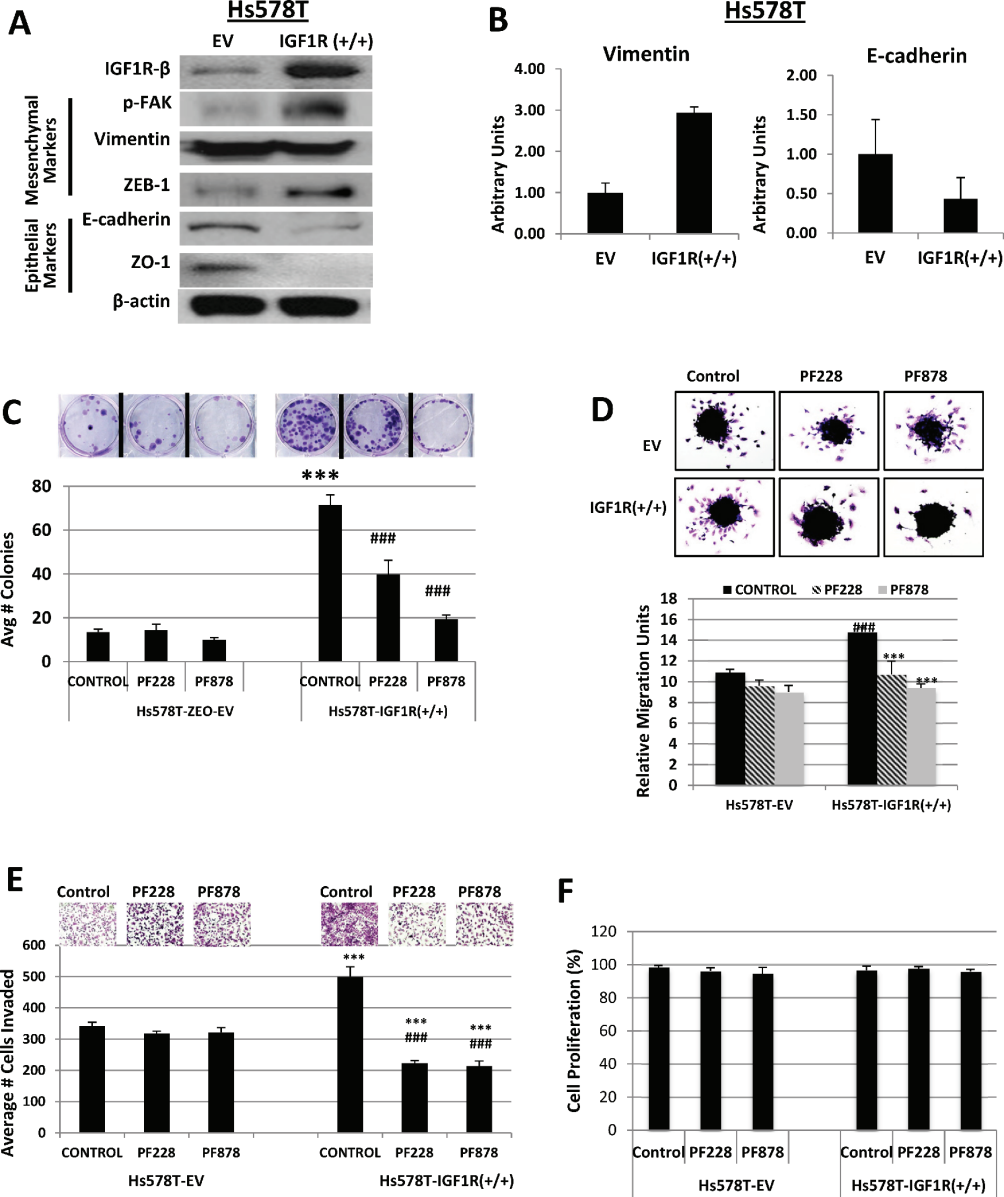
Inhibition of FAK abrogates IGF1R-mediated colony formation, migration, and invasion in TNBC cells **(A)** Lysates of Hs578T TNBC cells stably expressing empty vector (EV) control or IGF1R-β expression plasmid (IGF1R +/+) were immunoblotted with specific antibodies for IGF1R-β, p-FAK, Vimentin, ZEB-1, E-cadherin, ZO-1, and β-actin loading control. **(B)** Relative mRNA expression levels of Vimentin and E-cadherin in EV and IGF1R(+/+) TNBC cell lines were detected as described above. Data are displayed in means ± S.D. of at least three independent experiments of each group. **(C)** Colony formation, **(D)** spheroid migration, and **(E)** Matrigel invasion assays were performed as described above on Hs578T TNBC cells expressing either empty vector (EV) control or overexpressing full-length IGF1R-β (IGF1R +/+) followed by treatments with DMSO (control), 0.5 μM PF228, or 0.5 μM PF878 for 24 h. ***p < 0.001 compared to Hs578T-EV untreated controls; ^###^*p* < 0.001 compared to Hs578T-IGF1R(+/+) untreated cells. **(F)** Hs578T (EV) and Hs578T-IGF1R(+/+) cells were treated with 0.5 μM PF228 or 0.5 μM PF878 for 24 h and cells were counted by trypan blue exclusion. Data represented as a percentage of the vehicle treated (DMSO) control groups. The results represent the average of triplicated treatment groups performed at least three times with reproducible results.

Recent studies have implicated FAK overexpression as an important factor in cancer migration, invasion and metastasis, including breast cancers [29, 30], but its precise mechanisms of action remain unclear. Intriguingly, we found that abrogation of IGF1R expression in MDA-MB-231 and BT549 TNBC cells resulted in decreased FAK activation (Figure 1D). Although our findings clearly show the involvement of IGF1R in TNBC cell migration and invasion, we raised the question whether IGF1R-dependent cell migration and invasion requires FAK protein. We prepared Hs578T cells stably transfected with EV-control (Hs578T-EV) or full-length IGF1R expression plasmid (Hs578T-IGF1R(+/+)) to test the potential requirement of FAK in IGF1R-mediated migration and invasion. Immunoblotting analyses revealed that IGF1R overexpression increased FAK activity and ZEB-1 expression, which was accompanied by a decrease in the epithelial biomarkers E-cadherin and ZO-1 (Figure 6A). No observable changes were noted in vimentin expression, probably because these cells already express very high levels of the protein. Additionally, quantitative real-time PCR experiments showed that IGF1R overexpressing cells expressed lower E-cadherin and higher vimentin mRNA levels compared to IGF1R-null cells (Figure 6B).

We next analyzed the effects of FAK inhibition on IGF1R-mediated colony formation, migration, and invasive abilities of Hs578T-EV and IGF1R(+/+) cells using FAK-specific inhibitors, PF228 and PF878. In agreement with the absence of active FAK protein, Hs578T-EV cells were unresponsive to both PF228 and PF878 treatments; however, Hs578T-IGF1R(+/+) cells exhibited reduced clonogenecity following treatments with both inhibitors (Figure 6C). Likewise, FAK inhibitor treatments did not affect migration of IGF1R-null Hs578T cells, but IGF1R-overexpressing Hs578T cells displayed increased cell migration, which was inhibited in the presence of both FAK inhibitors (Figure 6D). Unlike IGF1R-null cells, which continued to invade in the presence of PF228 and PF878 inhibitors, IGF1R overexpression induced increased invasion of TNBC cells, which could also be effectively decreased by FAK inhibitors (Figure 6E). Inhibitor treatments had no observable effects on cell proliferation in either cell line (Figure 6F). Collectively, these results demonstrate that FAK is an integral molecule in mediating clonogenecity and the pro-migratory and pro-invasive effects of IGF1R in TNBC cells.

## DISCUSSION

**Figure 7:**
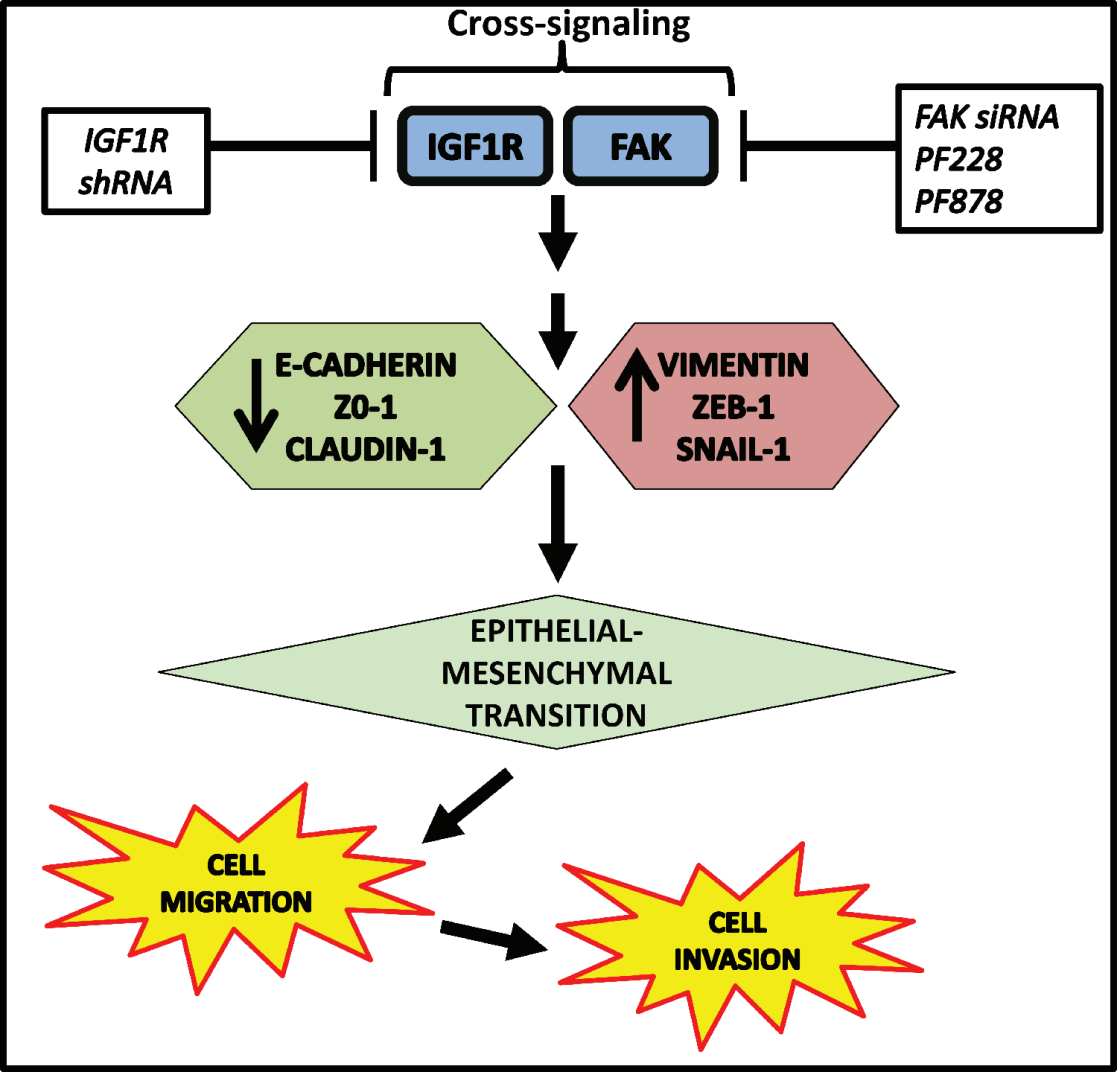
Proposed model of IGF1R/FAK crosstalk in TNBC IGF1R/FAK signaling increases expression of the mesenchymal markers (vimentin, ZEB-1, and Snail-1) and decreases expression of epithelial markers (E-cadherin, ZO-1, and claudin-1) with subsequent facilitation of EMT, leading to increased cell migration and invasion. The potential sites of therapeutic intervention are indicated and lead to decreased EMT, cell migration, and cell invasion.

Multiple studies have implicated a role for IGF1R in breast cancer, including TNBC, and other malignancies; however, the specific molecular mechanisms underlying IGF1R's involvement in TNBC are not fully understood. Our present study identifies a novel role for IGF1R signaling in the regulation of EMT and provides new insight into the downstream processes regulated by IGF1R in TNBC cells. This study is the first to show the cooperative effect of IGF1R and FAK signals in promoting malignant behaviors of TNBC cells. We propose a model of IGF1R/FAK crosstalk as one possible mechanism for regulating EMT, motility, and invasion in TNBC cells (Figure 7). A major finding from our study is that FAK-dependent IGF1R signaling is critical for maintaining mesenchymal morphologies and potentiating cell migration and invasion in TNBC cells. We found that inhibition of IGF1R induces changes resembling MET and decreases invasiveness in mesenchymal TNBC cells as evidenced by increased E-cadherin and decreased vimentin, ZEB1, and pFAK expression. Additionally, down-regulation of IGF1R significantly reduced colony number, invasive ability, and motility of TNBC cells, and ectopic expression of IGF1R produced opposite results.

Numerous cellular actions of the IGF-I/IGF1R signaling axis support its involvement in breast tumorigenesis, as it positively regulates cell survival, anti-apoptotic properties, and cell migration. Evidence from *in vitro* studies suggests that IGF1R signaling has differential effects on biological processes in ER-positive compared to ER-negative breast tumors. For example, IGF-I induces mitogenic and anti-apoptotic responses in ER-positive MCF-7 breast cancer cells; however, in ER-negative MDA-MB-231 cells, IGF-1 only positively affects cell motility [31]. Immunohistochemical studies of invasive breast tumor tissues showed that nearly 42% of TNBC patient samples expressed markedly high active IGF1R/IR levels, which was suggested to be indicative of poor survival [4]. Taken together, these observations and our current findings suggest that certain cellular functions of IGF1R are vital for the metastatic spread of ER-negative breast cancer cells.

EMT-like processes facilitate tumor progression and cancer metastasis, which is supported by clinical data showing that EMT regulators correlate well with tumor aggressiveness and poor patient outcomes [32]. Elevated vimentin and reduced E-cadherin levels correlate with increased breast cancer cell migration, invasion, and malignant cancer phenotypes [33]. ER-negative breast tumors express higher levels of vimentin than ER-positive tumors and *in vivo* expression of vimentin correlates strongly with more malignant breast tumor phenotypes [34]. Others and we have shown that 50–75% of TNBC breast cancers express vimentin, which is associated with high tumor grade, ER−/PR− status, and chemo-resistance [35, 36]. In addition, Lehmann et al. identified two mesenchymal-like subtypes of TNBCs [27], and another tissue-microarray based study examining 479 invasive breast carcinomas identified several EMT markers associated with basal-like breast tumors [37]. Vimentin expression in TNBCs is associated with younger age and poor prognosis [36]. Collectively, these and our data demonstrate that mesenchymal-like TNBCs are common and may be particularly prone to EMT and metastatic recurrence.

Several transcriptional regulators of EMT, including members of the ZEB, Snail, and Twist families, negatively control E-cadherin expression. Mounting evidence suggests that malignant breast cancer cells undergo EMT, especially TNBC subtypes [38]. ZEB1 mRNA levels are inversely associated with high-grade breast tumors and appear to be involved in tumor metastasis and poor patient survival [39]. Elevated ZEB1 expression has also been associated with metastatic pancreatic carcinomas [40]. Our findings are consistent with previous reports identifying ZEB1 as a downstream target of IGF1R, potentially assisting EMT processes in breast cancer cells [41–43].

Given the importance of IGF1R signaling in carcinogenesis, a number of IGF1R-specific pharmacological antagonists, including small molecule inhibitors and human monoclonal antibodies, are currently at various stages of preclinical and clinical investigations (reviewed in [11]). Available clinical trials have not produced encouraging results to date, perhaps because available agents do not effectively inhibit IGF1R signaling [44, 45]. Our findings indicate that IGF1R could be a unique molecular target, of particular relevance in mesenchymal TNBCs by demonstrating that IGF1R inhibition reduces the invasive characteristics of mesenchymal-like TNBC cell lines *in vitro*. These results provide the first characterization of the cellular effects of IGF1R/FAK inhibition on EMT in mesenchymal TNBC cells. Given the fact that a significant percentage of TNBCs express IGF1R, FAK, and mesenchymal markers [5, 36, 46], we consider our findings to have potentially important clinical implications for TNBC patients with mesenchymal phenotypes and warrant future clinical investigations which dually target the IGF1R and FAK signaling cascades.

## MATERIALS AND METHODS

### Reagents

The pLKO.1-IGF1R-α/β short hairpin RNA (shRNA) plasmid and pLKO.1 empty vector plasmid (negative control) were purchased from Open Biosystems (Huntsville, AL, USA). The pBABE-bleo-neo empty vector (plasmid 1766) and pBABE-bleo-IGF1R full-length expression vector (plasmid 11212) were obtained from Addgene (Cambridge, MA, USA). Dr. Adam Marcus at the Winship Cancer Institute of Emory University provided the pCMV-dR8.2 and pCMV-VSV-G helper constructs. RIPA cell lysis buffer was from Cell Signaling Technology, Inc. (Beverly, MA, USA), and protease inhibitor cocktail and phosphatase inhibitors were purchased from Sigma (Saint Louis, MO, USA). Puromycin was obtained from Invitrogen. PF573228 (PF228) was commercially available from Santa Cruz BioTechnology (Dallas, TX, USA) and PF04554878 (PF878) was generously provided by Dr. Adam Marcus. Matrigel and zeocin were purchased from BD Biosciences and agarose was from (Bio-Rad, Hercules, CA, USA).

### Cell culture

MDA-MB-231, BT549, and Hs578T cell lines were obtained from American Type Culture Collection (Manassas, VA, USA). Dr. Rita Nahta at the Winship Cancer Institute of Emory University generously provided the HEK-293T packaging cell line. These cell lines were not authenticated. Cells were routinely maintained in Dulbecco's Modification of Eagle's Medium (DMEM) supplemented with 10% Fetal Bovine Serum (FBS) and 2 μM L-glutamine (Invitrogen, Carlsbad, CA, USA).

### Lentivirus preparation

1.5 × 10^6^ HEK-293T cells were seeded in 100 mm dishes for 24 h and co-transfected with 3 μg shRNA constructs (pLKO.1-IGF1R-α/β shRNA (KD) or pLKO.1 empty vector (EV) control plasmids), 3 μg pCMV-dR8.2, and 0.3 μg pCMV-VSV-G helper constructs using TransIT-LT-1 Transfection reagent according to the manufacturer's instructions (Mirus Bio LLC, Madison, WI, USA). Forty-eight hours after transfections, viral stocks were harvested from culture media by centrifugation to remove cells and syringe-filtered. TNBC cell lines were seeded at sub-confluent densities and infected with lentiviruses (1:20 dilution) in fresh culture media. Culture media was replaced with media containing 2-μg/ml puromycin 48 hours after lentivirus infection to select for cells stably expressing the shRNAs (IGF1R-KD) or EV control plasmid. Stable clones were harvested after several passages for use and/or cryopreservation in liquid nitrogen.

### siRNA transient transfections

siRNA was used to silence the FAK gene. FAK siRNA: FAK siRNA-1, 5′-GUAUUGGACCUGCGAGGGA-3′ (sense) and 5′-UCCCUCGCAGGUCCAAUAC-3′ (antisense); FAK siRNA-2, 5′-CGAAUGAUAAGGUGU ACGA-3′ (sense) and 5′-UCGUACACCUUAUCAUU CG-3′ (antisense) were commercially available from Sigma-Aldrich (St Louis, MO, USA). A scrambled sequence siRNA (Control siRNA) was also used as a negative control (Sigma-Aldrich). Transit-TKO transfection reagent (Mirus Bio LLC, Madison, Wisconsin, USA) was used to optimize siRNA transfection according to the manufacturer's instructions. Briefly, Transit-TKO and siRNA were diluted separately in serum-free Opti-MEM media (Gibco) and incubated for 5 min at room temperature. The two solutions were gently mixed and incubated together for 30 min at room temperature. After incubation, the complex was added to the plated cells. Forty-eight hours post transfection, cells were further assayed in colony formation, spheroid migration, and Matrigel invasion assays as detailed below.

### Western blotting

Following treatments, total cell lysates were prepared using previously described methods [47]. Primary antibodies against pIGF1R (Tyr1135/1136), IGF1R-β, Snail-1, claudin-1, and ZO-1 were from Cell Signaling Technology, Inc. (Beverly, MA, USA). ZEB-1 antibody was from Santa Cruz Biotechnology, Inc. Antibodies against β-actin, and vimentin purchased from Sigma (Saint Louis, MO, USA) and pFAK (Tyr397), total FAK, and E-cadherin from BD Biosciences were used for Western blots according to standard protocol. Bound primary antibodies were detected with peroxidase-coupled secondary antibodies (Southern BioTech; Birmingham, AL, USA) and developed by enhanced chemiluminescence (Luminata Classico Western HRP substrate; EMD Millipore Corp.; Billerica, MA, USA).

### Real-time RT-PCR

Total RNA was extracted using the RNeasy purification kit (Qiagen, Valencia, CA 91355) and treated with DNase (Invitrogen). cDNA was prepared from total RNA using random primers and the Superscript III first strand synthesis Kit (Invitrogen). Relative levels of mRNA were determined by real-time quantitative PCR using an Eppendorf cycler and the TaqMan Universal PCR master Mix (Applied Biosystems, Carlsbad, CA). Primers for Vimentin (Hs00185584_m1), ZEB-1 (Hs-00232783_m1), E-cadherin (Hs01023894_m1), and RPLPO (Hs99999902_m1) were obtained from Applied Biosystems (TaqMan Gene Expression Assays). Samples were normalized against the RPLPO internal control using the 2–ΔΔCt method, compared as arbitrary units, and represented as mean ± SD. Samples were performed in triplicate and experiments were repeated at least 3 times with reproducible results.

### Colony formation assay

Single cell-suspensions were seeded in 12-well plates (250 cells/well) and allowed to grow. After 10 days, colonies (< 50 cells/colony) were fixed in 10% methanol and stained with crystal violet (0.1% in 20% methanol). Pictures were acquired using an Olympus Arcturus microscope (Mountain View, CA, USA), Infinity-2 Analyze digital camera, and software (Lumenera Corp., Ottawa, ON, Canada).

### Matrigel invasion chamber assay

Invasion assays were performed using Transwell chambers with 8-μm pore polycarbonate membrane inserts (Corning Inc., Corning, NY, USA). 50,000 cells/well were plated onto the upper chambers Matrigel-coated inserts and allowed to invade for 24 hours. Fetal bovine serum and fibronectin were used in the lower chambers as chemoattractants. Non-invading cells were removed and invaded cells in the membrane were fixed in methanol, washed, and stained with 0.1% crystal violet. Inserts were washed, briefly air-dried, and mounted. Invaded cells were counted using an inverted microscope (20X magnification). Five fields were counted for each sample.

### Spheroid migration assay

For migration assays, 2.0 × 10^4^ cells suspended in complete medium containing 2.5% reconstituted basement membrane (rBM, Matrigel) were seeded onto 1.0% agar-coated 96-well plates and cultured for 48 hours in a humidified atmosphere containing 5% CO_2_ at 37°C. Intact tumor spheroids were carefully transferred to six-well plates and cultured in complete media for 24–48 hours. Spheroids and migrated cells were fixed with 10% buffered formalin, stained with 0.05% crystal violet, and observed using a normal light microscope (20X) and Olympus DP-30BW digital camera. The distance of migration from the center of the spheroid was quantified using Image Pro software and is representative of three independent experiments, each performed in triplicate.

### Stable overexpression of IGF1R

Human Hs578T TNBC cells were grown to 70% confluency and transfected with 3.0 μg control plasmid (pBABE-bleo) or IGF1R expression vector (pBABE-bleo-IGF1R) using Transit-LTI according to manufacturer's instructions. Forty-eight hours post transfection, culture media was refreshed with media containing 100 μg/ml of zeocin antibiotic to select for clones stably expressing the control vector and IGF1R expression vector. Stable cell lines were used for subsequent Western blotting analyses, colony formation, spheroid migration, and Matrigel invasion assays as described above.

### Trypan blue exclusion

For growth inhibition assays, cells were plated in complete DMEM at 2 × 10^4^ cells/per well in 12-well plates. The next day, media were aspirated and replaced with media containing vehicle control or FAK inhibitors in triplicate. After 24 h, viable cells were counted under a light microscope by trypan blue exclusion. Assays were repeated at least three times with reproducible results.

### Cell survival assay

Cells were seeded at a density of 5000 cells/well in 96-well plates and grown overnight before treatment with control and FAK siRNAs or indicated concentrations of PF228 and PF878 in complete culture media for 24 hours. Cell viability was assessed using sulforhodamine-B (SRB) assays following procedures previously described (27).

### Statistical analysis

Quantitative data from *in vitro* experiments are presented as mean ± SD of experiments repeated at least three times in triplicate. Differences among group means were analyzed using one-way ANOVA or unpaired Student's *t*-test. Differences were considered significant at *p* < 0.05.
